# Direct Detection of Fungal Siderophores on Bats with White-Nose Syndrome via Fluorescence Microscopy-Guided Ambient Ionization Mass Spectrometry

**DOI:** 10.1371/journal.pone.0119668

**Published:** 2015-03-17

**Authors:** Samantha J. Mascuch, Wilna J. Moree, Cheng-Chih Hsu, Gregory G. Turner, Tina L. Cheng, David S. Blehert, A. Marm Kilpatrick, Winifred F. Frick, Michael J. Meehan, Pieter C. Dorrestein, Lena Gerwick

**Affiliations:** 1 Center for Marine Biotechnology and Biomedicine, Scripps Institution of Oceanography, University of California San Diego, La Jolla, CA 92093, United States of America; 2 Department of Chemistry and Biochemistry, University of California San Diego, La Jolla, CA, United States of America; 3 Skaggs School of Pharmacy and Pharmaceutical Sciences, University of California San Diego, La Jolla, CA, United States of America; 4 Pennsylvania Game Commission, 2001 Elemerton Ave., Harrisburg, PA 17110, United States of America; 5 Department of Ecology and Evolutionary Biology, University of California Santa Cruz, Santa Cruz, CA, United States of America; 6 United States Geological Survey, National Wildlife Health Center, 6006 Schroeder Road, Madison, WI 53711, United States of America; University of South Dakota, UNITED STATES

## Abstract

White-nose syndrome (WNS) caused by the pathogenic fungus *Pseudogymnoascus destructans* is decimating the populations of several hibernating North American bat species. Little is known about the molecular interplay between pathogen and host in this disease. Fluorescence microscopy ambient ionization mass spectrometry was used to generate metabolic profiles from the wings of both healthy and diseased bats of the genus *Myotis*. Fungal siderophores, molecules that scavenge iron from the environment, were detected on the wings of bats with WNS, but not on healthy bats. This work is among the first examples in which microbial molecules are directly detected from an infected host and highlights the ability of atmospheric ionization methodologies to provide direct molecular insight into infection.

## Introduction

Fungal diseases of animals and plants have long been a feature of natural ecosystems, but evidence suggests that incidences of mycoses are increasing in frequency and severity and that these emerging infectious diseases pose a threat in terms of loss of biodiversity and food security [1]. Mycoses have garnered a large amount of attention recently due to their detrimental impacts on populations of organisms as varied as sea fans, turtles, bees, corals, frogs, crayfish, and bats [1–7]. The fungal infection affecting hibernating North American bats, white-nose syndrome (WNS), has killed an estimated 5.7–6.7 million bats since it was first described in 2006 and has spread to 25 states and five Canadian provinces [8–11]. The psychrophilic fungus identified as the causative agent of WNS, *Pseudogymnoascus destructans*, is believed to be an exotic pathogen of European origin that was introduced to the U.S. through human activity [12–17].

The mechanism through which WNS induces mortality remains to be fully elucidated. It is hypothesized that cutaneous infection of bat wing skin with *P*. *destructans* disrupts electrolyte balance [18–19]. This imbalance or some other mechanism causes increases in the frequency with which infected bats arouse from torpor during winter [19]. Frequent arousals may in turn deplete fat stores and lead to death by starvation [20].

Identifying molecules that fungal pathogens use to interact with a host provides insight into the mechanism of their pathogenicity. Better understanding these mechanisms may lead to improved management strategies that account for the biology of the pathogen. We therefore set out to directly profile the metabolic milieu on the wings of bats with WNS with a focus on fungal molecules. We used fluorescent microscopy-guided ambient mass spectrometry to metabolically profile the wings of bats of the genus *Myotis* that were healthy (n = 5) or showed signs of WNS infection (n = 11). This will enable us to begin to establish a ‘molecular signature’ for WNS and to determine if there are molecules that the fungus may use to facilitate infection.

## Materials and Methods

### Animal Collection & Tissue Sample Preparation

Five healthy *Myotis lucifugus*, 10 *M*. *lucifugus* with WNS, and one *Myotis septentrionalis* with WNS were included in the analysis (Table 1). All bats were found deceased during routine monitoring of maternity or hibernation roosts being conducted by state agency biologists. In Pennsylvania, personnel of the Pennsylvania Game Commission collected the specimens in compliance with Pennsylvania Statute Title 34, Section 322. Deceased bats collected in West Virginia were collected by personnel of the West Virginia Department of Natural Resources and no permits were required. Healthy bats were collected from 2004 to 2008 in Pennsylvania. All but one of these bats were collected prior to the emergence of WNS in North America and the bat collected after WNS emergence had no obvious signs of *P*. *destructans* infection (e.g. cupping erosions) when evaluated microscopically. WNS bats were collected in 2011 in Pennsylvania and in West Virginia during a WNS-associated mass mortality event. No bats were euthanized for this study. Two 6 mm tissue punches were collected from the wings of each of the deceased bats, affixed to 1x3 inch microscope glass slides, and stored at -80°C prior to mass spectrometric analysis.

**Table 1:** Specimen information for the *Myotis* used in the study.

WNS Status	Species	Sex	Collection Date	Collection Location
Pre-WNS	*Myotis lucifugus*	unknown	11/12/08	Barton Cave, Fayette County, PA
Pre-WNS	*Myotis lucifugus*	female	8/15/05	Shaver’s Creek, Huntington County, PA
Pre-WNS	*Myotis lucifugus*	male	9/13/04	Harlansburg Cave Gate, Lawrence County, PA
Pre-WNS	*Myotis lucifugus*	female	4/16/05	Canoe Creek Mine, Blair County, PA
Pre-WNS	*Myotis lucifugus*	female	4/16/05	Canoe Creek Mine, Blair County, PA
WNS	*Myotis lucifugus*	female	2/26/11	Hellhole Cave, Pendelton County, WV
WNS	*Myotis lucifugus*	female	2/26/11	Hellhole Cave, Pendelton County, WV
WNS	*Myotis lucifugus*	male	2/26/11	Hellhole Cave, Pendelton County, WV
WNS	*Myotis lucifugus*	male	2/26/11	Hellhole Cave, Pendelton County, WV
WNS	*Myotis lucifugus*	male	2/26/11	Hellhole Cave, Pendelton County, WV
WNS	*Myotis lucifugus*	female	2/26/11	Hellhole Cave, Pendelton County, WV
WNS	*Myotis lucifugus*	female	2/26/11	Hellhole Cave, Pendelton County, WV
WNS	*Myotis lucifugus*	male	2/26/11	Hellhole Cave, Pendelton County, WV
WNS	*Myotis lucifugus*	male	2/26/11	Hellhole Cave, Pendelton County, WV
WNS	*Myotis lucifugus*	male	2/26/11	Hellhole Cave, Pendelton County, WV
WNS	*Myotis septentrionalis*	unknown	1/7/11	Lincoln Cavern, Huntington County, PA

### Microscopy Ambient Ionization Mass Spectrometry of Bat Wings

**Fig 1:**
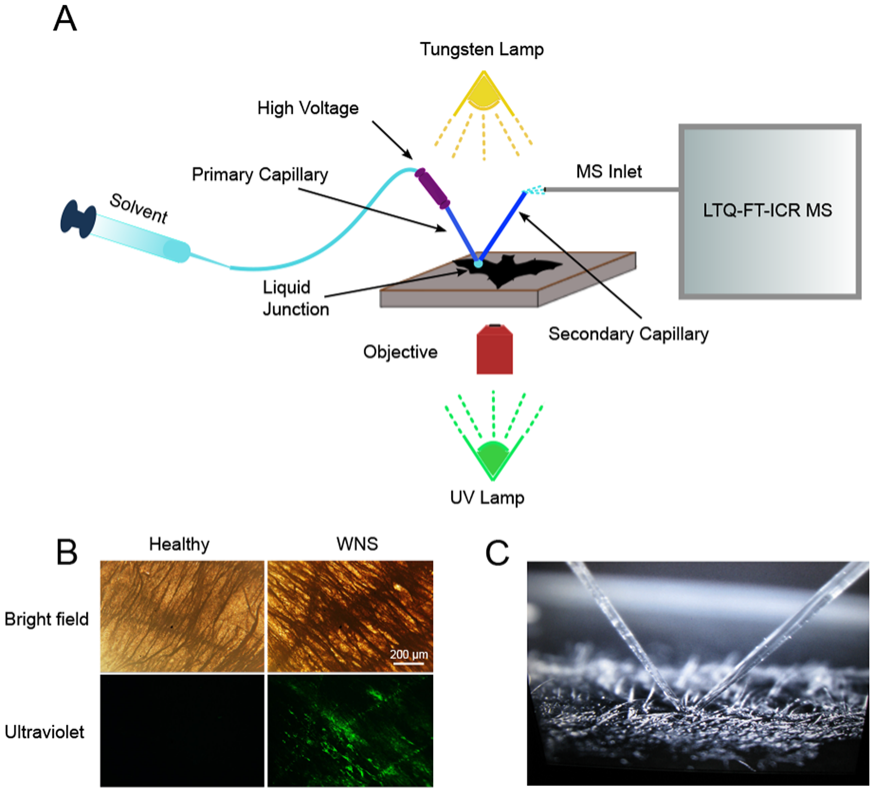
Microscopy ambient ionization mass spectrometry of bat wings. Tissue samples from the wings of healthy bats and individuals with white-nose syndrome were subjected to microscopy ambient ionization mass spectrometry (A). *Pseudogymnoascus destructans* infection was confirmed by the presence of fluorescent lesions when the tissue was excited with UV light (B). A nanoDESI source was used to desorb analytes from fluorescent tissue regions for MS analysis (capillary junction making contact with the tissue surface, C).

The mass spectrometric interrogation of bat wing skin was performed using a hybrid microscopy/ionization technique which combines an ambient nanospray desorption electrospray ionization (nanoDESI) source and an inverted microscope as described [21] (Fig. 1A). Unlike previous usage of these tools, our analysis incorporated the use of fluorescence. This allowed us to illuminate the wings with UV light and visualize areas of fluorescence that correlate with the development of cupping erosions due to infection with *P*. *destructans* [22] (Fig. 1B). We could then directly target these areas with the nanoDESI probe for MS analysis (Fig. 1C). The sample slide with wing punches was placed on the stage of a Nikon DIAPHOT 300 microscope and bright field and fluorescent images of the tissue were captured using a CCD camera (Nikon D40 DSLR) to confirm the presence or absence of fungal infection. The stage was then manipulated to move the tissue sample to the desired position under the micrometer-sized liquid junction formed by the two flame-pulled fused silica capillaries of the nanoDESI. The capillary tubes were flamed-pulled from original 150/50 μm (O.D./I.D.) to ~60 μm O.D. and a voltage of 2.2 kV was applied to them throughout the experiment. The capillaries were aligned in a “V” configuration so that they abutted one another at the bottom of the “V” and then angled 45° away from the point of contact in either direction. A syringe pump was used to continuously deliver the solvent (either acetonitrile in water (65/35, vol/vol) with 0.2% formic acid, methanol in water (50/50, vol/vol) with 0.2% formic acid, or methanol, acetonitrile, and toluene (50/35/15, vol/vol/vol) with 0.2% formic acid) at a rate of **~**1.0 μL/min through 300/100 μm (O.D./I.D.) fused silica capillary tubing to the primary flame-pulled capillary. This solvent was then aspirated by the secondary capillary resulting in the formation of a dynamic liquid droplet of approximately 100 μm in diameter at the junction of the two capillaries. The sample stage was raised until the tissue contacted the liquid droplet and a continuous stream of solvent containing analytes desorbed from the tissue was delivered to the inlet of the mass spectrometer (hybrid 6.4-T LTQ-FT; Thermo Electron, North America) via electrospray ionization generated at the terminal end of the secondary capillary. Multiple spots were sampled along each tissue slide.

### Fungal Extraction for MS Analysis

Replicates of *P*. *destructans* were grown on ISP2 agar plates (4 g/liter yeast extract, 10 g/liter malt extract, 4 g/liter dextrose, and 1.5 to 2% agar). Small plugs of agar and fungal biomass were collected and combined with 100 μl of *n*-butanol in microcentrifuge tubes. Extractions were allowed to proceed at room temperature for one hour. The organic layers were centrifuged and the supernatant was subsequently analyzed. A Triversa nanomate-electrospray ionization source was used to introduce the extracts (diluted in methanol in water (50:50) with 1% formic acid) directly into the MS inlet. This was accomplished using a spray voltage setting of 1.3–1.45 kV and a pressure of 0.35–0.5 psi as set with Chipsoft software version 7.2.0. Data were collected using Xcalibur software version 1.4 SR1 running the data-dependent method described below.

### Mass Spectrometric Analysis

A 6.4 T Finnigan LTQ-FT-ICR MS (Thermo Electron, North America) was used to analyze tissue samples and fungal extracts. The instrument was tuned to 816 *m/z* using bovine cytochrome *c* (charge state 15, Sigma Aldrich) in a 65/35 (vol/vol) acetonitrile/0.2% formic acid aqueous solution with Tune Plus software version 1.0. Ion spectra from 150–2,000 *m/z* were collected in positive ion mode. In cases where there was a high background, the ion spectra observation range was reduced. The automated instrument scan cycle consisted of two segments. The first segment had two profile mode MS^1^ scans: one full scan in the IT mode with 200 ms max fill time; one full scan in the FT cell (50,000 resolution) with 8 sec max inject time. This was followed by an MS^1^-dependent tandem mass (MS/MS) acquisition which consisted of 9 scans in a cycle with a Δ*m/z* = 3 isolation window. The top 9 most abundant peaks in the MS^1^ scan were sequentially isolated and fragmented by applying collision induced dissociation (CID) energy using nitrogen as the collision gas. Ion peaks that had been selected twice were excluded and not fragmented again in subsequent cycles in order to cover as many individual ions as possible. These data-dependent MS/MS scans (IT mode, profile spectra) consisted of a maximum 500 ms fill time, 35% normalized collision energy, 0.25 activation Q, and 0.05 s activation time. Each scan contained 4 microscans and was recorded in average. Data were acquired for 10 minutes.

### Molecular Networking

The structural relationships between different masses in MS^1^ scans (precursor ions) were mapped in Cytoscape version 2.8.3 based on the similarity of their tandem MS fragmentation patterns as assessed through molecular networking using the web-based Global Natural Products Social Molecular Networking tool (GNPS; http://gnps.ucsd.edu/)[23–25]. A cosine cutoff of 0.6 was selected for generation of the network. Background signals arising from solvents or agar were subtracted during the network processing stage. The network was then imported into Cytoscape for visualization. Precursor ions were represented by nodes and the similarity between two precursor ions as determined by comparison of their fragments/cosine score was represented as an edge between two nodes. The magnitude of the cosine score was represented by edge thickness with pairs of compounds with high scores having thicker lines. A search of the publicly curated GNPS standards library was also performed to determine if any of the masses represented in the network matched to known compounds that we had not considered in our analysis. All data files used in the generation of the network were deposited in the GNPS MassIVE data repository and are publicly available (MassIVE ID MSV000078620).

### Seeding the Network with Desferrichrome, Ferrichrome, and Triacetylfusarinine C Standards

Iron-free ferrichrome (Santa Cruz Biotechnology), ferrichrome (Iron-free ferrichrome with the addition of iron (III) chloride hexahydrate), and triacetylfusarinine C (EMC microcollections GmbH) were used as standards to verify the molecular assignment of the siderophores in the tissue samples through a process known as dereplication [25]. The solubilized standards were introduced directly into the MS inlet with a Triversa nanomate-electrospray ionization source and analyzed using the data-dependent method described above. A molecular network was then generated using the data files from the standards, fungal extracts, and tissue samples. The nodes correlated with the standard data files were examined to establish if they (1) clustered with experimental nodes or (2) were incorporated into consensus nodes that also included experimental files. Following this neighborhood analysis, the spectra were manually examined and the fragmentation of the raw spectra evaluated for similarity. Through this analysis it was possible to definitively determine whether or not desferrichrome, ferrichrome and triacetylfusarinine C were detected from the wing surfaces and/or from fungal cultures.

## Results and Discussion

### Detection of fungal metabolites on bat wings by fluorescence microscopy ambient ionization mass spectrometry and molecular networking

**Fig 2:**
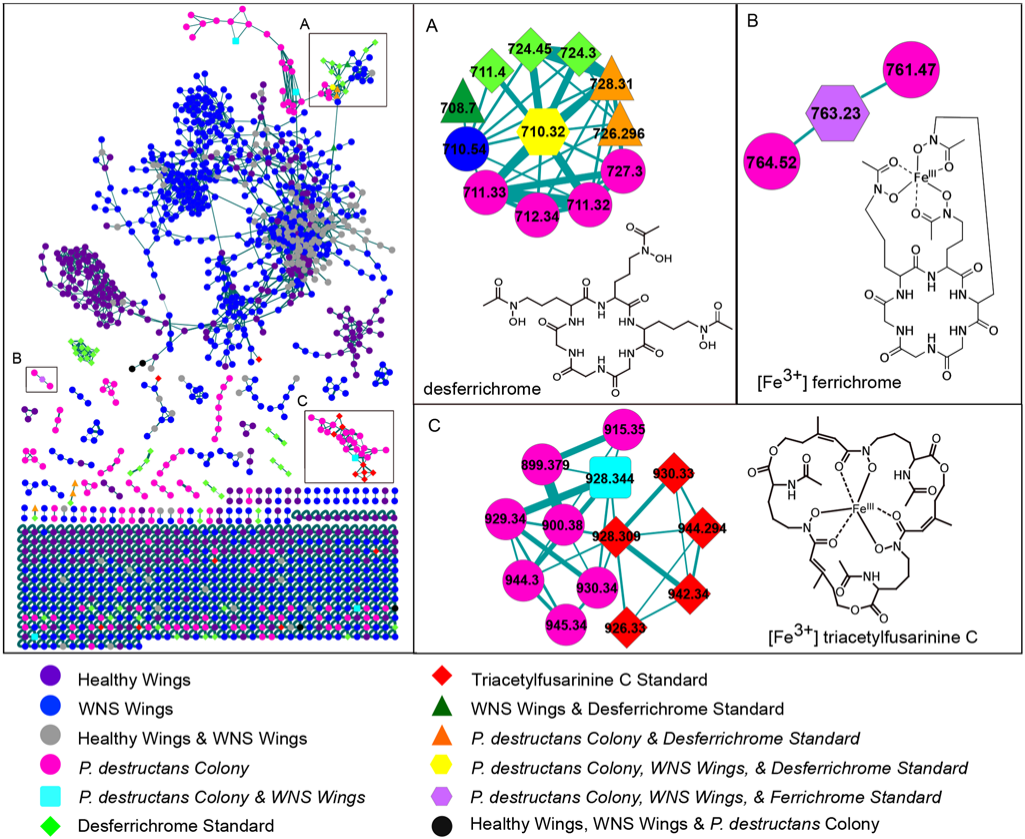
Detection of siderophores on the wings of bats with white-nose syndrome via molecular networking. Molecular networking of the MS/MS data was used to determine the structural relationships between the metabolites detected from wing surfaces, cultured *Pseudogymnoascus destructans*, and commercial siderophore standards. The siderophores desferrichrome (*m/z* 710.324) and ferrichrome (*m/z* 763.230) were observed from the wings of bats with white-nose syndrome and from cultured *P*. *destructans* and formed consensus nodes with commercial standards (A,B). Triacetylfusarinine C (*m/z* 928.344) was also present on WNS wings and in the *P*. *destructans* colony (C). None of the three siderophores were detected on the wings of healthy bats. In A and C insets only the node with the *m/z* of interest and its first and second neighbors have been displayed from the overall cluster for simplicity.

Fluorescent microscopy ambient mass spectrometry of bat wings, extracts from cultured *P*. *destructans*, and commercial standards generated a large number of MS/MS spectra. These spectra were subjected to molecular networking [23,25] giving rise to a network comprised of 1,503 nodes representing precursor ions derived from intact chemical compounds and 2,463 edges representing their structural relationships (Fig. 2A; S1 Fig.). Each node was colored with respect to the sample type(s) from which it derived. The resulting molecular network provides a visual picture of the diversity and origin of MS/MS spectra that were collected.

The network highlighted a number of molecules that were present on the wings of bats with WNS that were not detected from healthy bats and *vice versa*. These distinct chemical signatures were found to be due to the presence of fungal metabolites on the WNS wings. Individual variation and a host response to infection likely also contribute the observed difference. Several of the MS/MS spectra from the experimental samples matched those of known fungal metabolites and these nodes were inspected in further detail.

In the first node we inspected, spectra from fungal extracts matched to a compound in the GNPS standards library, the mycotoxin citrinin, indicating that P. destructans may have the capacity to produce citrinin or a citrinin-like molecule (S2A, S2B Fig.). This skin-permeable nephrotoxin is known to be produced by fungi of the genera *Penicillium*, *Monascus*, and *Aspergillus* [26–27]. A protein blast of the *P*. *destructans* genome using the citrinin polyketide synthase from *Monascus purpureus* as the query revealed a homologous sequence with 44% identity. (S2C Fig.). It may be interesting to evaluate the overall ability of the fungus to produce mycotoxins in the future and to evaluate the contributions of any such molecules to pathogenicity.

**Fig 3:**
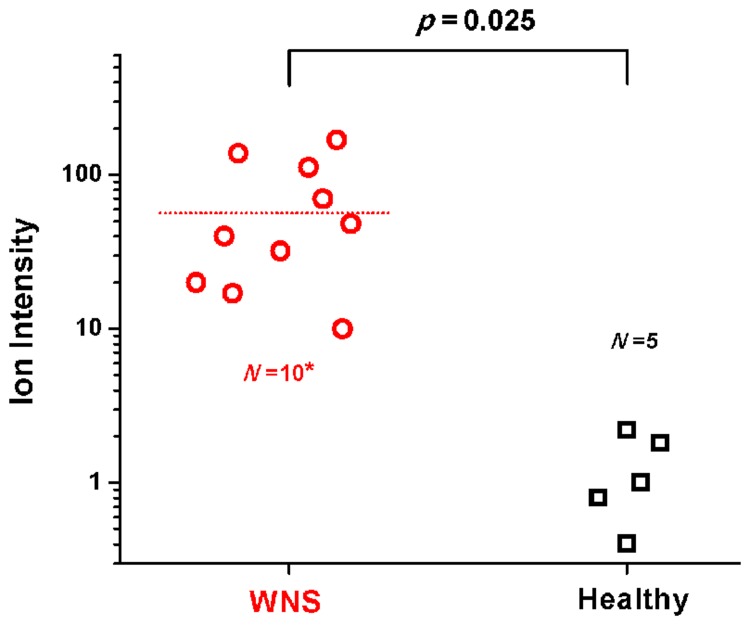
Statistical analysis of desferrichrome daughter ion intensity among healthy and diseased bat wings. Analysis of variance (ANOVA) of the absolute intensities of the desferrichrome daughter ion, *m/z* 650 (neutral loss of C_2_H_4_O_2_), observed on WNS and healthy bat wings (See also S4 Fig.). A ± 3 *m/z* ion window was allowed when selecting the precursor, *m/z* 710. Differences in the absolute intensities of the daughter ions among 10 of the 11 WNS wings and 5 healthy wings were statistically significant (*p* = 0.025). On the eleventh WNS wing, no ions within the *m/z* 710 ± 3 *m/z* range was selected for fragmentation by the automatic data-dependent method due to low ion intensity and the sample could therefore not be included in the plot.

Another node was a match for the fungal siderophore desferrichrome (*m/z* 710.324, Fig. 2A). Siderophores are microbial iron-chelating molecules that are either retained intracellularly as a means of iron storage or are of a diffusible type that gather ferric iron from the extracellular environment and are then retrieved by the organism [28]. The node incorporated scans from WNS wings, the cultured *P*. *destructans* colony, and the desferrichrome standard; scans from the wings of healthy bats were not included (Fig. 2A). Manual inspection of the MS/MS fragmentation data for these precursors confirmed that the pattern observed from the WNS wings and the fungal colony matched that of the commercial desferrichrome standard (S3 Fig.). This indicates that the masses observed from the cultured fungus and on the wing skin of infected bats derive from desferrichrome and that, presumably, *P*. *destructans* is producing this siderophore during its colonization of bats. When the signal for the siderophore was analyzed in a targeted fashion, by performing MS/MS directly on the isolated parent ion during microscopy nanoDESI analysis, desferrichrome was detected on 10 of the 11 WNS bats and was virtually undetectable in all of the samples from healthy bat wings (ANOVA, *p* = 0.025; Fig. 3; S4 Fig.). In addition, the iron-chelated form of the molecule, ferrichrome, was detected (*m/z* 763.230, Fig. 2B; S5 Fig.). This indicates that there is sufficient iron present on bat wings to be chelated by the siderophore.

A second siderophore, triacetylfusarinine C, was also detected by molecular networking (Fig. 2C). A node with *m/z* 928.344 incorporated scans from a WNS wing and the cultured *P*. *destructans* colony and clustered with triacetylfusarinine C standard nodes. Manual inspection of the tandem mass spectra confirmed that the node derived from the fragmentation of triacetylfusarinine C, although it failed to form a consensus node with the standard under the molecular networking parameters selected (S6 Fig.). This indicates that this siderophore is also produced by *P*. *destructans* during infection.

Interrogation of the *P*. *destructans* genome for secondary metabolite enzymes involved in siderophore biosynthesis and imaging mass spectrometry of the cultured *P*. *destructans* colony further support the production of ferrichrome and triacetylfuarinine C by the fungus (S1 Supporting Information; S7 Fig.; S1 Table). In the absence of a controlled laboratory experiment in which bats are analyzed before and after infection with *P*. *destructans*, it cannot be unequivocally stated that the observed metabolites are produced by *P*. *destructans* since other fungi also have the ability to form these siderophores. However, this seems unlikely given the marked difference we observe between WNS and healthy wings and we hypothesize that *P*. *destructans* requires iron in order to grow and mount infection and therefore produces siderophores during its colonization of bats.

Microbes require iron as a cofactor in redox reactions involved in a variety of cellular processes. Consequently, a large part of the host response to infection centers on limiting the amount of iron available to invading microbes [29–32]. Because of this, the amount of available iron is, in general, sub-optimal for microbial growth [30,33]. Whether this is the case for hibernating bats is unknown.

High-affinity siderophores are important for satisfying microbial iron demand in the face of host defenses and a number of investigations have demonstrated that they are essential for growth and virulence [34–38]. In addition, production of hydroxamate siderophores is a feature of the dermatophytic pathogenic fungi *Microsporum* and *Trichophyton* and ferrichrome-type siderophores, specifically, have been demonstrated to be necessary for epithelial invasion by *C*. *albicans* in an *in vitro* model of oral candidiasis [39–40]. Our detection of fungal siderophores on wings of bats with WNS suggests that these molecules may have a role in infection and/or tissue invasion.

Because of the importance of iron in microbial infections, a number of antimicrobial strategies that target siderophores have recently emerged. These include efforts that link antibiotics or antifungals to siderophores to mediate their uptake, inundation with irrelevant siderophores that the organism cannot use or analogues that cannot bind iron, addition of iron chelators such as EDTA, and disruption of endogenous siderophore pathways through the blocking of biosynthetic enzymes or transporters [41–44]. We contend that such methods of iron deprivation should be investigated for their effectiveness against *P*. *destructans* infection.

## Conclusion

Here we used fluorescence microscopy ambient mass spectrometry in combination with molecular networking to detect metabolites directly from the wing skin of bats with WNS. The approach established the presence of fungal siderophores on infected bat wings. Siderophores are important for fungal growth and virulence and methods that interfere with their biosynthesis or activity may prove effective in limiting *P*. *destructans* infection.

## Supporting Information

S1 Table Comparison of known siderophore biosynthetic enzymes to putative *Pseudogymnoascus destructans* proteins. (XLSX)

S1 Fig Molecular network composition. The molecular network was composed of nodes that incorporated MS/MS scans from only a single sample type as well as consensus nodes that incorporated spectra from different experimental sample types. The number of nodes in each category and their relationships to each other are conveyed in a Venn diagram.
(TIF)

S2 Fig Potential for production of the mycotoxin citrinin by *Pseudogymnoascus destructans*. A comparison of the data to a library of standards from the Global Natural Products Social Molecular Networking database resulted in a putative match, citrinin, to the *P*. *destructans* culture extract (A,B). A protein blast of the *P*. *destructans* genome using the polyketide synthase responsible for citrinin biosynthesis as the query returned a homologous amino acid sequence (C).
(TIF)

S3 Fig Comparison of desferrichrome fragmentation among standards and samples. MS/MS of the desferrichrome standard *m/z* 710 (A). The MS/MS fragmentation patterns from the *P*. *destructans* colony (B) and WNS wing (C) matched the fragmentation of the standard precursor.
(TIF)

S4 Fig Variation in the intensity of desferrichrome daughter ion *m/z* 650 detected from white-nose syndrome wing samples. In cases where desferrichrome precursor ions display high intensities relative to other metabolites, their fragmentation patterns will be less complicated and they will have a greater cosine correlation with the standard (A). When the intensities of the desferrichrome ions are less intense, background noise or peaks from other compounds of similar mass may be fragmented along with them resulting in more complicated MS/MS spectra and a lower cosine correlation with the standard even though the molecule is present (B).
(TIF)

S5 Fig Comparison of ferrichrome fragmentation among standards and samples. FT-MS/MS of ferrichrome Fe^3+^ complex standard *m/z* 763 (A). The IT-MS/MS fragmentation patterns for *m/z* 763 from the *P*. *destructans* colony (B) and WNS wings (C) matched the fragmentation of the standard precursor.
(TIF)

S6 Fig Comparison of triacetylfusarinine C fragmentation among standards and samples. FT-MS/MS of triacetylfusarinine C Fe^3+^ complex standard *m/z* 928 (A). The IT-MS/MS fragmentation patterns for *m/z* 928 from the *P*. *destructans* colony (C) and WNS wings (D) matched the fragmentation of the standard precursor.
(TIF)

S7 Fig Imaging mass spectrometry of *Pseudogymnoascus destructans* colonies. *P*. *destructans* was grown directly on ISP2 agar (Ai) or on top of a permeable membrane overlaid on agar (Aii) which was later removed for IMS analysis. Colonies and agar were excised and placed on a MALDI target plate along with a portion of plain agar which served as a negative control (B). Aerial hyphae were removed from the *P*. *destructans* colony grown directly on the agar, and the membrane and *P*. *destructans* colony were removed from the second sample to reveal the agar underneath (C). Several metabolites were observed to be associated with the fungal colony (Di) or secreted into the agar underneath it (Dii). Among the metabolites for which ions were observed were the siderophores desferrichrome and triacetylfusarinine C.
(TIF)

S1 Supporting Information Supporting Methods and Results for Imaging Mass Spectrometry and Genomic Analysis. (PDF)
