# Supplementary figures and images for: Direct Detection of Fungal Siderophores on Bats with White-Nose Syndrome via Fluorescence Microscopy-Guided Ambient Ionization Mass Spectrometry
Source: PLoS One. 2015 Mar 17;10(3):e0119668. doi: 10.1371/journal.pone.0119668 (PMC4364562; doi:10.1371/journal.pone.0119668)

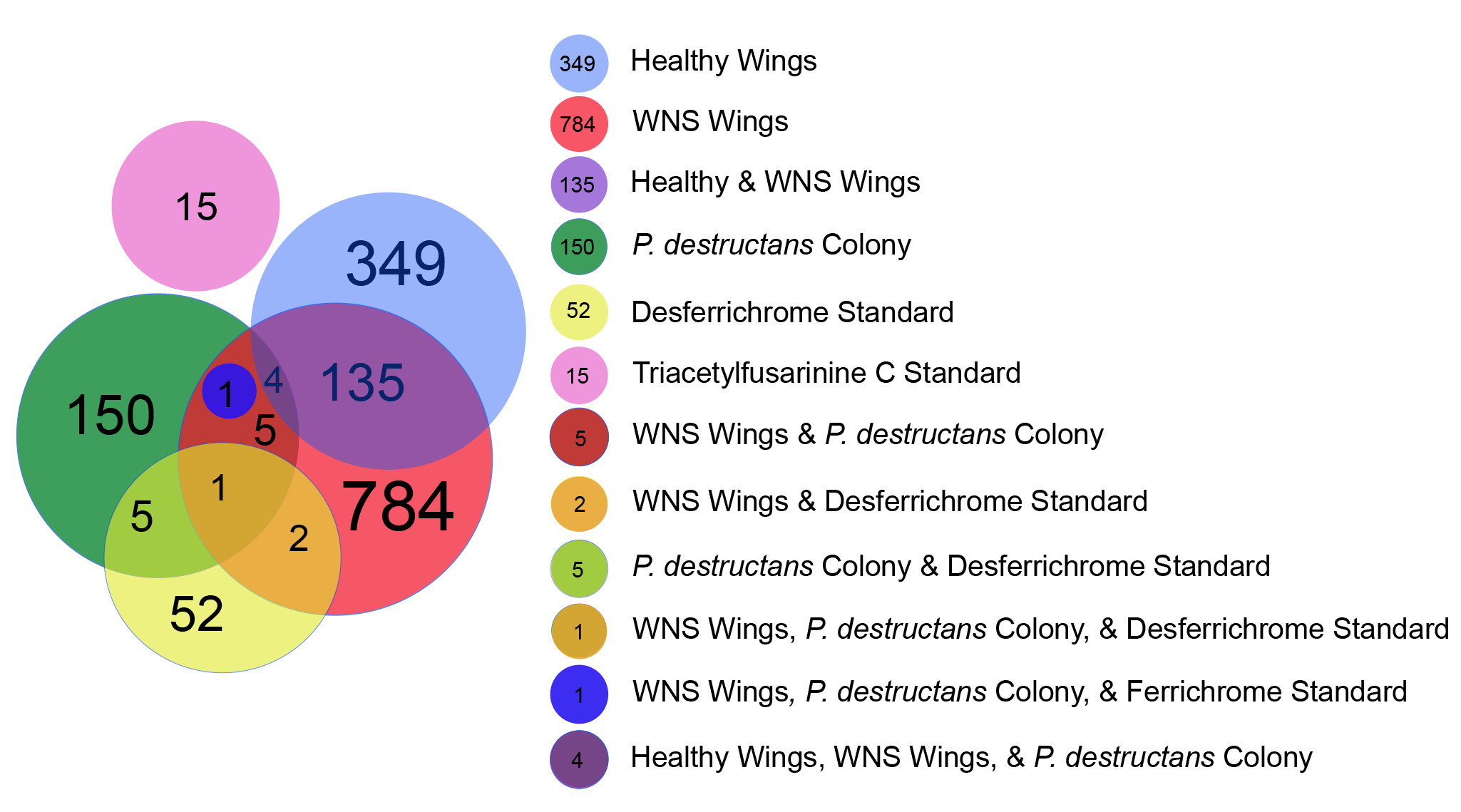

Supplement: S1 Fig — The molecular network was composed of nodes that incorporated MS/MS scans from only a single sample type as well as consensus nodes that incorporated spectra from different experimental sample types. The number of nodes in each category and their relationships to each other are conveyed in a Venn diagram. (TIF) [file pone.0119668.s002.tif]

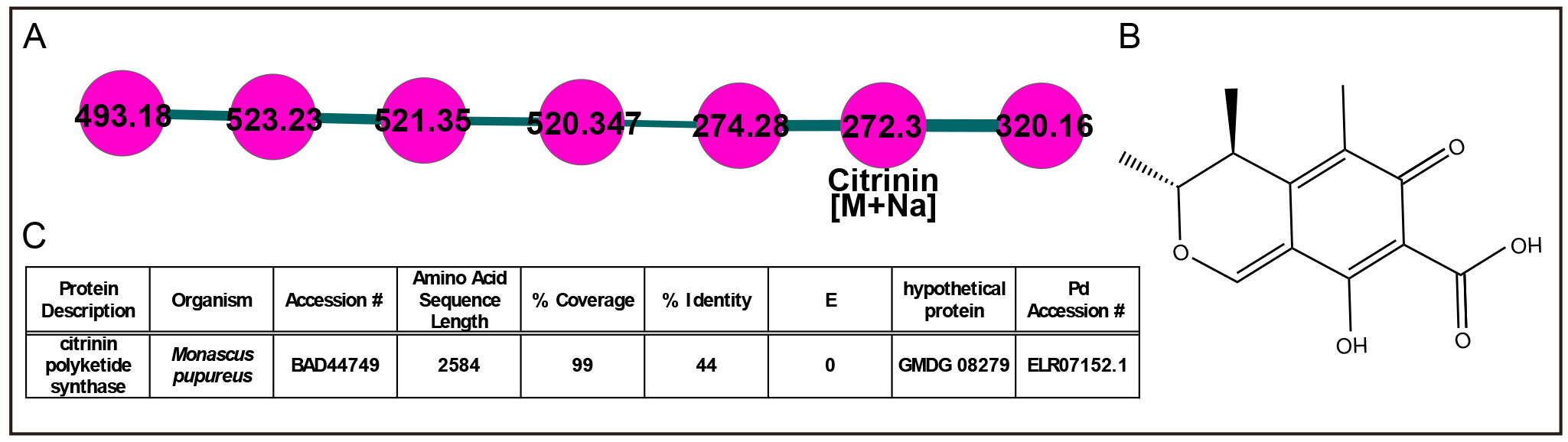

Supplement: S2 Fig — A comparison of the data to a library of standards from the Global Natural Products Social Molecular Networking database resulted in a putative match, citrinin, to the P. destructans culture extract (A,B). A protein blast of the P. destructans genome using the polyketide synthase responsible for citrinin biosynthesis as the query returned a homologous amino acid sequence (C). (TIF) [file pone.0119668.s003.tif]

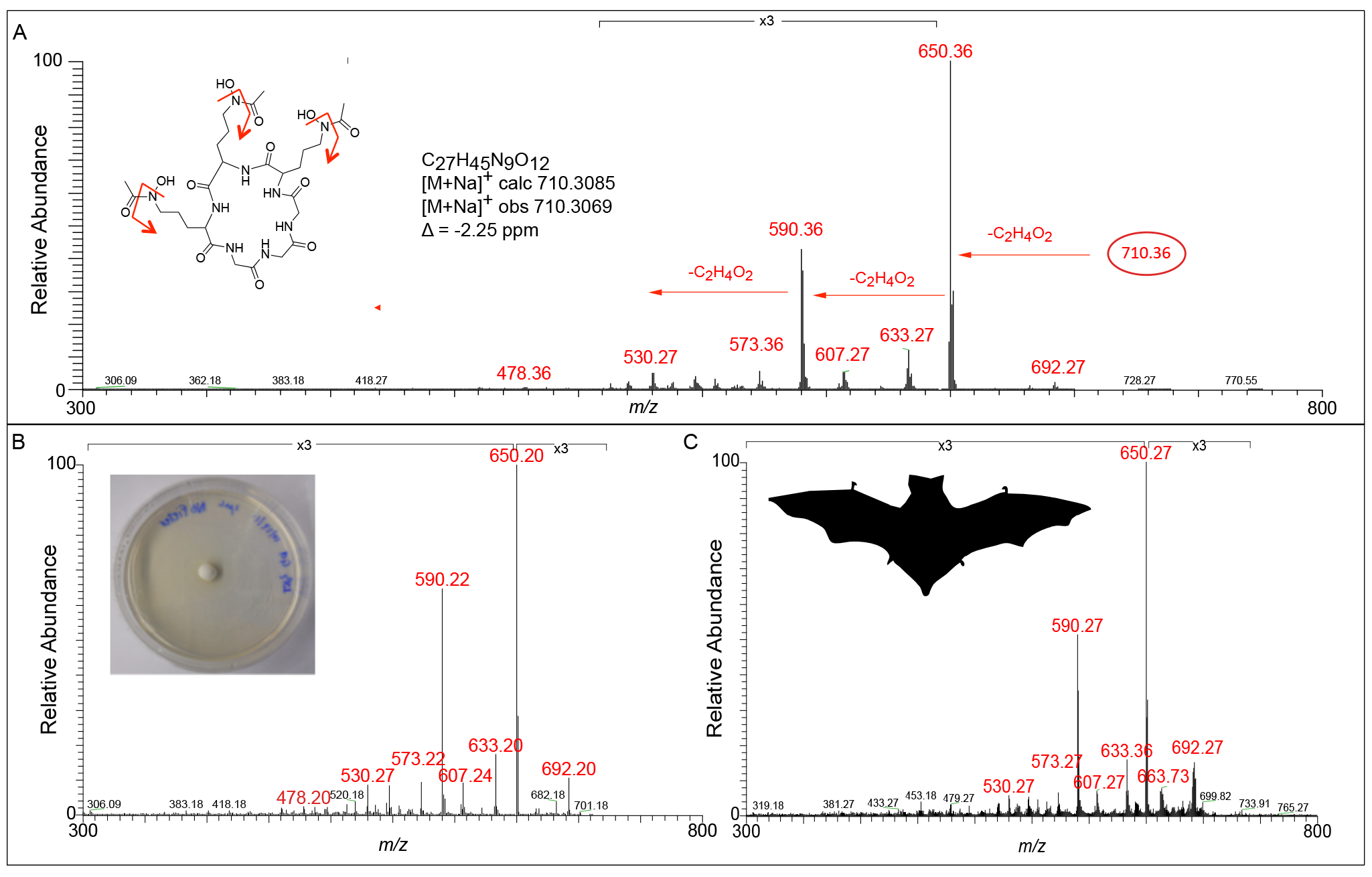

Supplement: S3 Fig — MS/MS of the desferrichrome standard m/z 710 (A). The MS/MS fragmentation patterns from the P. destructans colony (B) and WNS wing (C) matched the fragmentation of the standard precursor. (TIF) [file pone.0119668.s004.tif]

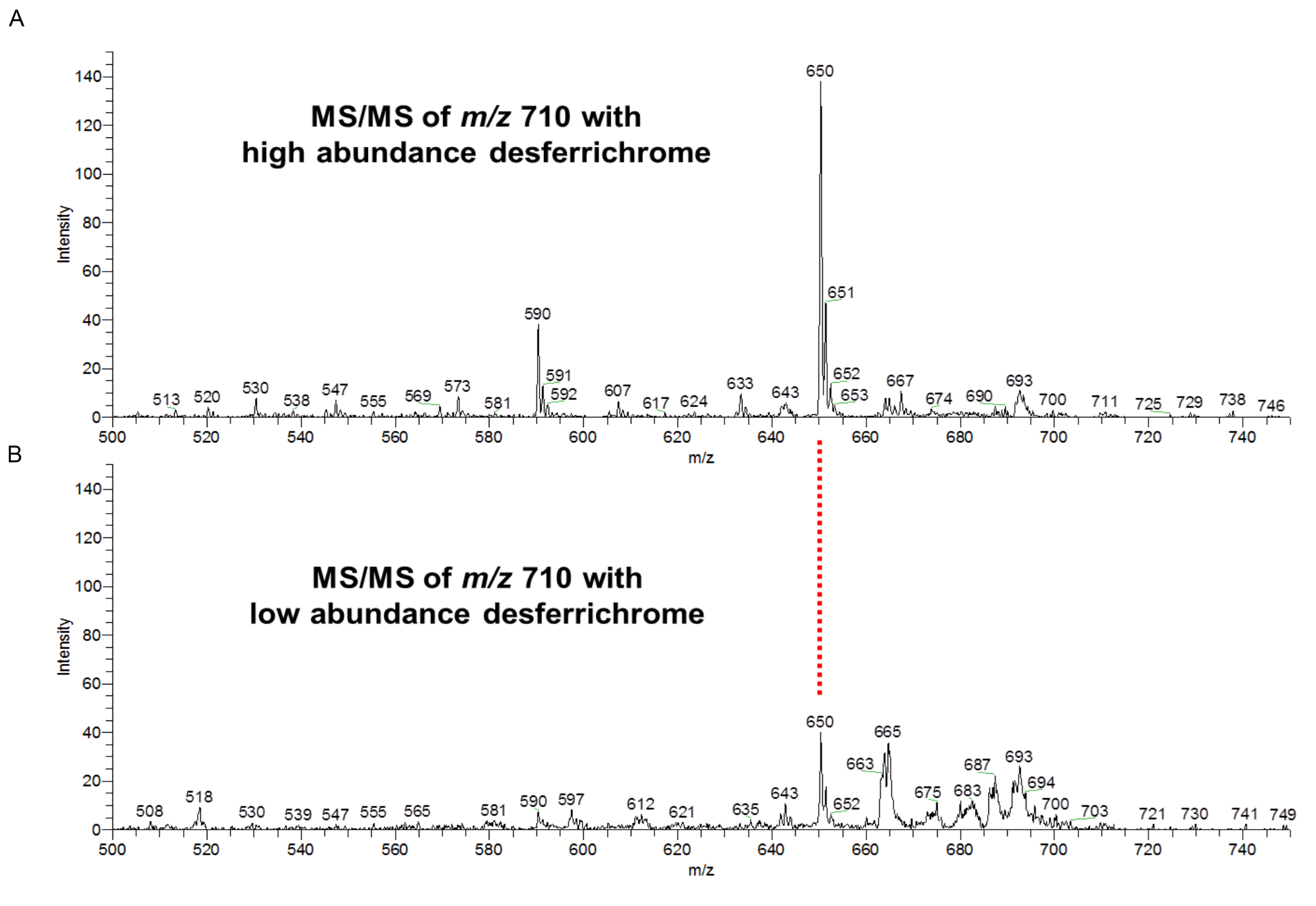

Supplement: S4 Fig — In cases where desferrichrome precursor ions display high intensities relative to other metabolites, their fragmentation patterns will be less complicated and they will have a greater cosine correlation with the standard (A). When the intensities of the desferrichrome ions are less intense, background noise or peaks from other compounds of similar mass may be fragmented along with them resulting in more complicated MS/MS spectra and a lower cosine correlation with the standard even though the molecule is present (B). (TIF) [file pone.0119668.s005.tif]

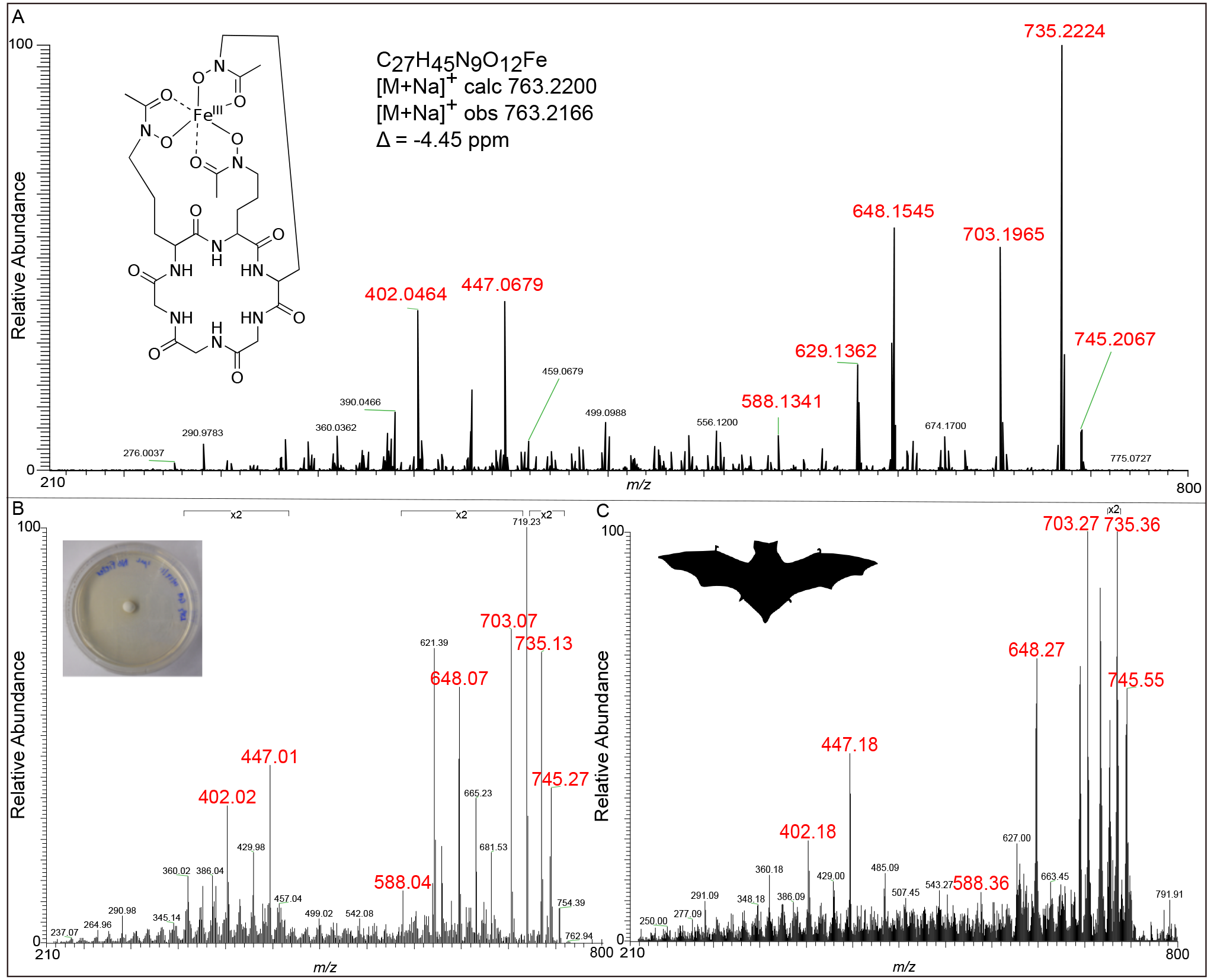

Supplement: S5 Fig — FT-MS/MS of ferrichrome Fe3+ complex standard m/z 763 (A). The IT-MS/MS fragmentation patterns for m/z 763 from the P. destructans colony (B) and WNS wings (C) matched the fragmentation of the standard precursor. (TIF) [file pone.0119668.s006.tif]

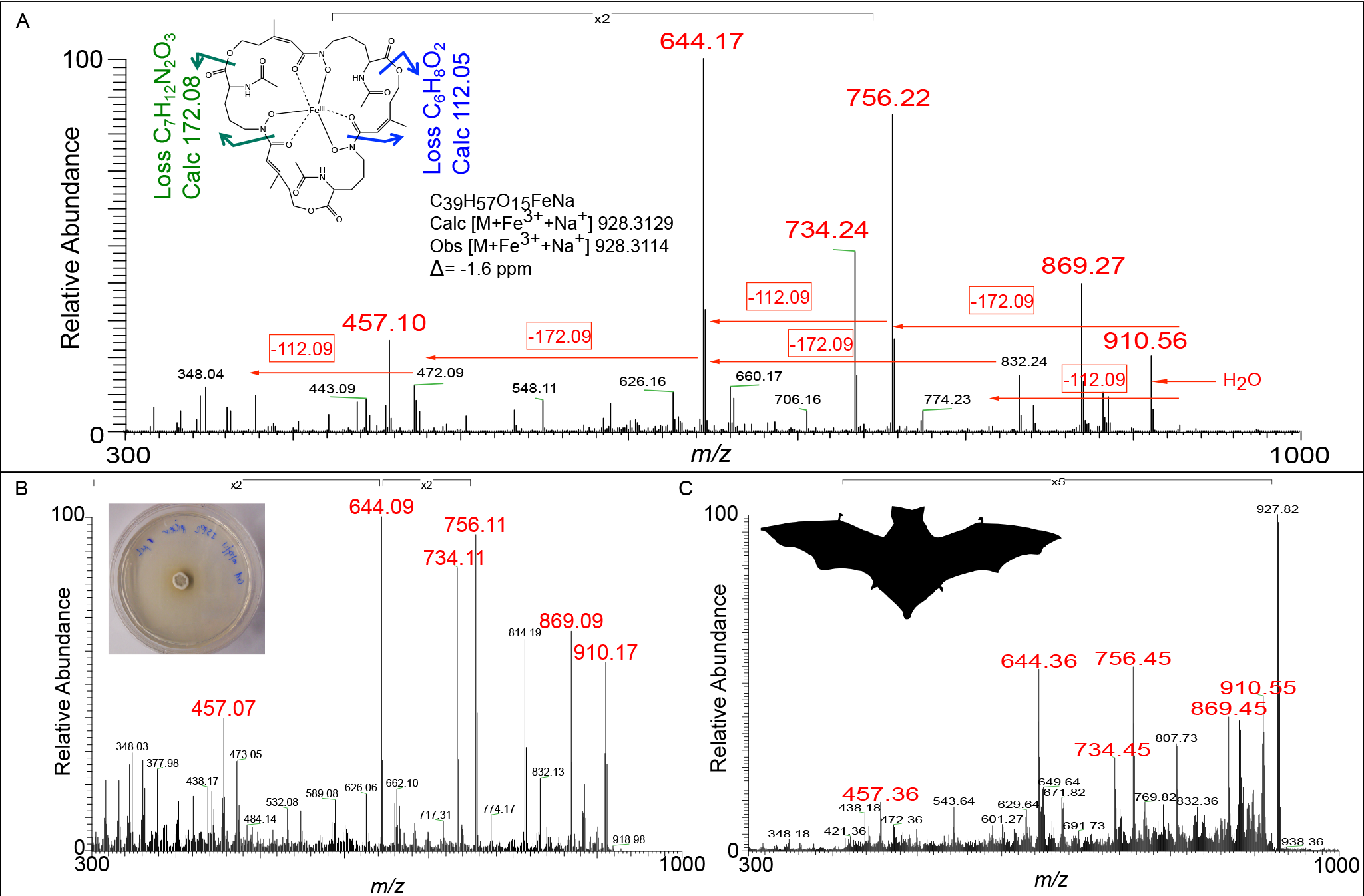

Supplement: S6 Fig — FT-MS/MS of triacetylfusarinine C Fe3+ complex standard m/z 928 (A). The IT-MS/MS fragmentation patterns for m/z 928 from the P. destructans colony (C) and WNS wings (D) matched the fragmentation of the standard precursor. (TIF) [file pone.0119668.s007.tif]

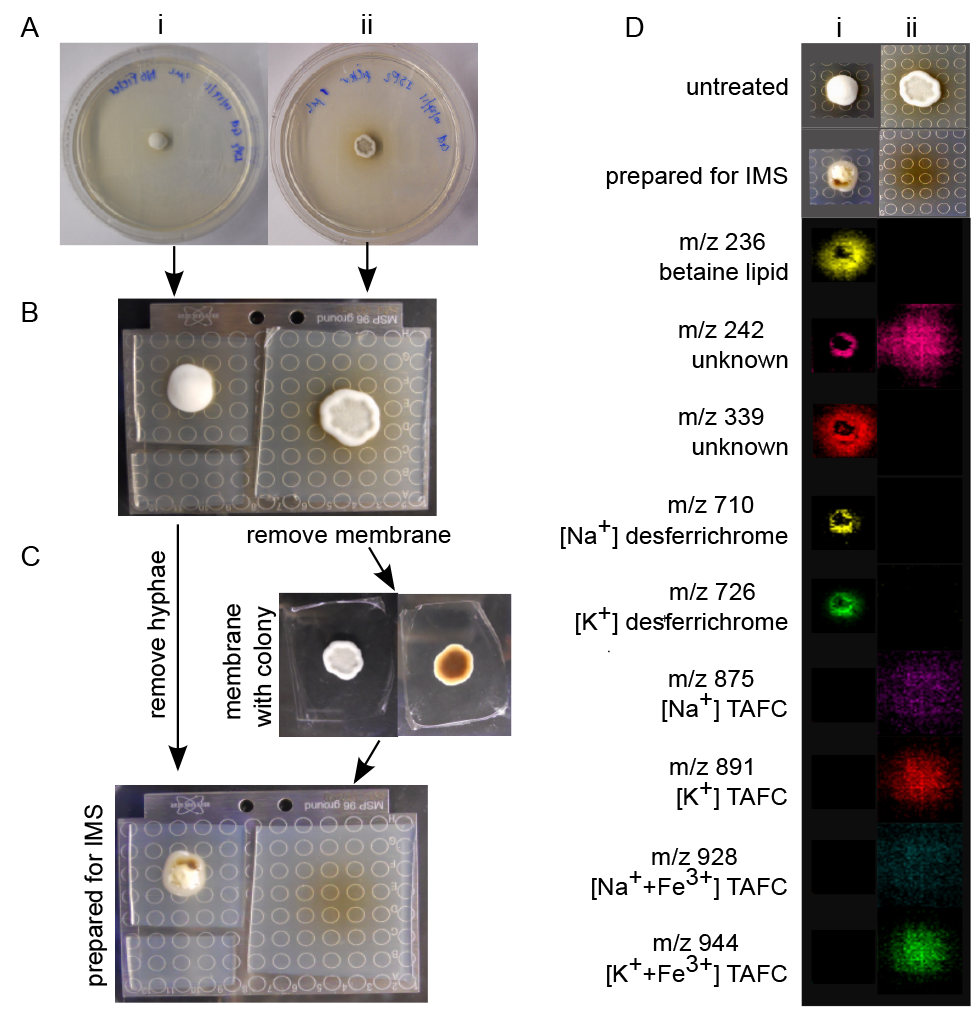

Supplement: S7 Fig — P. destructans was grown directly on ISP2 agar (Ai) or on top of a permeable membrane overlaid on agar (Aii) which was later removed for IMS analysis. Colonies and agar were excised and placed on a MALDI target plate along with a portion of plain agar which served as a negative control (B). Aerial hyphae were removed from the P. destructans colony grown directly on the agar, and the membrane and P. destructans colony were removed from the second sample to reveal the agar underneath (C). Several metabolites were observed to be associated with the fungal colony (Di) or secreted into the agar underneath it (Dii). Among the metabolites for which ions were observed were the siderophores desferrichrome and triacetylfusarinine C. (TIF) [file pone.0119668.s008.tif]
