# Supplementary material for: Direct Detection of Fungal Siderophores on Bats with White-Nose Syndrome via Fluorescence Microscopy-Guided Ambient Ionization Mass Spectrometry
Source: PLoS One. 2015 Mar 17;10(3):e0119668. doi: 10.1371/journal.pone.0119668 (PMC4364562; doi:10.1371/journal.pone.0119668)
Supplement: S1 Supporting Information — (PDF) [file pone.0119668.s009.pdf]

Supporting Information

Supporting Methods for Imaging Mass Spectrometry and Genomic Analysis

Growth and Preparation of Fungal Samples for MALDI-TOF Analysis

Materials. All chemicals used for ISP2 media and the Universal MALDI matrix were purchased from Sigma-Aldrich [1]. Organic solvents were purchased from J. T. Baker. Sabouraud-dextrose agar petri dishes containing chloramphenicol and gentamycin were purchased from BD diagnostic Systems through Fisher Scientific. Cellophane membranes were a gift from the Dr. Paul Straight lab. (Texas A&M).

Cultures of *P. destructans* were initiated from a starter culture of type isolate 20631-21 as designated by Gargas et al. [2]. Following incubation of an initial culture on Sabouraud Dextrose agar containing chloramphenicol and gentamycin at 11°C for 56 days, new cultures were started by aseptically transferring small pieces of the mycelial mass to fresh plates of both Sabouraud-dextrose agar containing chloramphenicol and gentamycin and ISP2 agar. These cultures were incubated at 11°C for 42 days after which time they exhibited a gray coloration indicating that conidia had formed. A spore stock solution was then prepared by addition of sterile water (700 μL) to the top of each colony grown on Sabouraud followed by re-aspiration and dilution with an equal volume of a 40% glycerol solution. The spore stock solution was stored at -78°C and used for subsequent inoculations.

Colonies of *P. destructans* for MALDI-TOF analysis were grown under two conditions. In the first, cellophane membranes were autoclaved in water, then placed on top of yeast extract/malt extract ISP2 agar (10 ml) in 100 x 25 mm Petri dishes and any air bubbles were removed [1]. *P. destructans* spore solution (1 μL) was then inoculated

onto these cellophane membrane-covered ISP2 agar plates. In the second condition, *P. destructans* spore solution was inoculated directly onto ISP2 agar plates lacking a membrane. Inoculates were inverted and incubated at 10 °C for 21 days. Two regions of ISP2 agar containing *P. destructans* colonies, one grown under each condition, were cut out to yield sections ranging from 1 x 2 cm to 3 x 4 cm and placed on top of a steel MALDI MSP 96 anchor plate (Bruker Daltonics, Billerica, MA). A small piece of fungus-free ISP2 agar was also placed on the same MALDI plate as negative control. A photograph of the sample plate was captured and then the membrane with conidia was removed from the piece of agar grown in the first condition, leaving only the agar itself on the target plate. The aerial hyphae from the other colony grown directly on ISP2 were gently removed with a cotton swab dampened in acetonitrile [3]. A second photograph was taken and a layer of Universal MALDI matrix (1:1 mixture of 2,5-dihydroxybenzoic acid and α-cyano-4-hydroxy-cinnamic acid) was applied to the sample using a 53 μm sieve. Samples were incubated at 37 °C for a minimum of 5 hr until completely dry and adhered to the MALDI plate, and were then analyzed by MALDI-TOF.

General MS Procedures for MALDI Sample Analysis.

The sample MALDI plate was subjected to MALDI-TOF mass spectrometry (Microflex from Bruker Daltonics, Billerica, MA) for IMS acquisition and was run in positive reflectron mode, with 550 μm laser intervals in XY and a mass range of 100-3000 Da. The data were analyzed using FlexImaging 2.0 software. Detailed instrument parameters for collecting image data were described previously [1,4].

Bioinformatic interrogation of *P. destructans* genome for biosynthetic enzymes

The web-based protein Basic Local Alignment Search Tool (pBLAST) available through the National Center for Biotechnology Information was used to search the genome of *P. destructans* (accession number AEFC00000000.1) for secondary metabolite enzymes [5-6]. The accession numbers for proteins reported from other organisms to be involved in ferrichrome and triacetylfusarinine C biosynthesis were entered into the query field of a Standard Protein BLAST. These were searched against the non-redundant protein sequences (nr) of *P. destructans*. Similarity in hypothetical *P. destructans* proteins was reported as percentage coverage, percentage identity, and the Expect value (E).

Supporting Results for Imaging Mass Spectrometry and Genomic Analysis

Imaging Mass Spectrometry of *P. destructans* cultured on agar

When matrix-assisted laser desorption ionization time of flight imaging mass spectrometry (MALDI-TOF IMS) of the *P. destructans* colony was performed, over 30 metabolites were observed that displayed a variety of distributions (Figure S7). Desferrichrome, *m/z* 710 and *m/z* 726, was clearly associated with the mycelial mass and was not detected underneath the colony indicating that it is present intracellularly. Although ferrichrome has been observed to be excreted by *Ustilago maydis*, this observation is in accordance with reports concerning other fungi in which ferricrocin-related siderophores, such as ferrichrome, are intracellularly formed and localized [7-10]. The Fe^{3+} chelated form of ferrichrome, however, was not unequivocally detected in the 2D-IMS. Another set of ions *m/z* 875, 891, 906, 928 were only detected in the agar

underneath the fungal colony. MS^2 network analysis of extracts indicated that these were the siderophore triacetylfusarinine C, present in desferri form (sodium and potassium adduct) as well as Fe-chelated forms (sodium and potassium adducts). As was found here for *P. destructans*, these siderophores are present in many other fungi and are known to be extracellularly excreted [7-8]. The detection of desferrichrome and triacetylfusarinine C in the colony lends support to our hypothesis that these compounds are being produced by *P. destructans* on bat wing skin.

Interrogation of the *P. destructans* genome for the presence of biosynthetic secondary metabolite enzymes and siderophore transporters

Interrogation of the *P. destructans* genome revealed evidence for the presence of biosynthetic secondary metabolite enzymes reported from other organisms to be responsible for production of ferrichrome and triacetylfusarinine C (Table S1) [7]. Homologous sequences were also discovered for the membrane transporters MirB and Sit1 which, in *Aspergillus nidulans,* internalize triacetylfusarinine C and ferrichrome respectively. These results further indicate that *P. destructans* has the capability to generate and internalize both of these hydroxamate siderophores.

References:

1. Yang JY, Phelan VV, Simkovsky R, Watrous JD, Trial RM (2012) Primer on Agar-Based Microbial Imaging Mass Spectrometry. J Bacteriol 194: 6023-6028.

2. Gargas A, Trest MT, Christensen M, Volk TJ, Blehert D (2009) *Geomyces destructans* sp. nov., associated with Bat White-Nose Syndrome. Mycotaxon 108:

147-154.

3. Moree WJ, Phelan VV, Wu CH, Bandeira N, Cornett DS, et al. (2012) Interkingdom metabolic transformations captured by microbial imaging mass spectrometry. Proc Natl Acad Sci *USA* 109: 13811-13816.

4. Yang YL, Xu YQ, Straight P, Dorrestein PC (2009) Translating metabolic exchange with imaging mass spectrometry. Nat Chem Biol 5: 885-887.

5. Altschul SF, Gish W, Miller W, Myers EW, Lipman DJ (1990) Basic local alignment search tool. J Mol Biol 215: 403-410.

6. Chibucos MC, Crabtree J, Nagaraj S, Chaturvedi S, Chaturvedi V (2013) Draft genome sequences of human pathogenic fungus *Geomyces pannorum* sensu lato and bat white nose syndrome pathogen *Geomyces (Pseudogymnoascus) destructans*. Genome Announc 1(6): e01045-13.

7. Haas H, Eisendle M, Turgeon BG (2008) Siderophores in fungal physiology and virulence. Annu Rev Phytopathol 46: 149-187.

8. Schrettl M, Bignell E, Kragl C, Sabiha Y, Loss O, et al. (2007) Distinct Roles for Intra- and Extracellular Siderophores during *Aspergillus fumigatus* Infection. PLoS Pathog 3(9): e128. doi:10.1371/journal.ppat.0030128

9. Charlang G, Ng B, Horowitz NH, Horowitz RM (1981) Cellular and extracellular siderophores of *Aspergillus nidulans* and *Penicillium*. Mol Cell Biol. 1(2): 94–100.

10. Winkelmann G (2007) Ecology of siderophores with special reference to the fungi. Biometals 20: 379-392.
